# Reduced Predictive Performance of the FIB‐4 Index in Chronic Hepatitis B Patients With Concurrent Metabolic Dysfunction‐Associated Liver Disease

**DOI:** 10.1111/jvh.70071

**Published:** 2025-09-08

**Authors:** Randa Taher Natour, Motti Haimi, Rawi Hazan, Tarek Saadi, Fadi Abu Baker

**Affiliations:** ^1^ Technion Israel Institute of Technology Rappaport Faculty of Medicine Haifa Israel; ^2^ Department of Gastroenterology and Hepatology Hillel Yaffe Medical Center Hadera Israel; ^3^ Health System Management Department The Yezreel Valey Academic College D.N Emek Yezreel Israel; ^4^ Liver Clinic, Clalit Health Services North District (Nazareth) Israel; ^5^ Azrieli Faculty of Medicine Bar‐Ilan University Safed Israel; ^6^ Liver Unit Rambam Health Care Campus Haifa Israel

**Keywords:** APRI score, chronic hepatitis B (CHB), cirrhosis, elastography, FIB4 score, fibrosis, metabolic‐associated steatotic liver disease (MASLD)

## Abstract

The coexistence of chronic hepatitis B (CHB) and metabolic dysfunction–associated liver disease (MASLD) gained recognition, but the diagnostic performance of non‐invasive markers regarding it remains underexplored. This study aimed to evaluate the utility of the FIB‐4 index for fibrosis prediction in CHB patients and investigate its performance in the distinct subgroup of CHB‐MASLD. A prospective study from 2021 to 2022 included 109 CHB and 64 CHB‐MASLD patients. All underwent liver stiffness measurement via elastography and FIB‐4 calculation. MASLD criteria were defined, creating CHB‐alone and CHB‐MASLD groups. FIB‐4 values were compared to the liver stiffness measurements. Statistical analyses included *t*‐tests, ROC curves, and correlation assessments. No significant age, gender, or ethnicity differences were observed between the CHB and CHB‐MASLD groups. CHB‐MASLD patients exhibited higher BMI, dysglycemia, and dyslipidemia. HBeAg negativity and nucleoside/tide treatment rates were comparable. FIB‐4 and APRI scores were elevated in CHB‐MASLD. ROC analysis revealed an AUC of 0.86 for FIB‐4 in the CHB group, with an optimal cutoff of 1.95. Subgroup analysis by BMI showed consistent FIB‐4 performance. In the CHB‐MASLD group, the ROC curve showed an AUC of 0.61 (*p* = 0.12), indicating non‐significance. Our study affirms FIB‐4's robust performance in predicting fibrosis in CHB patients across varied BMI profiles. Yet, challenges surface when applying FIB‐4 to those with concurrent CHB and MASLD. These limitations stress the urgency of refined fibrosis prediction tools, essential for navigating the complex interplay of viral and metabolic factors in the CHB–MASLD population.

**Abbreviations**
BMI: body mass index
CHB: chronic hepatitis B
FIB‐4: fibrosis‐4 index
HBA1C: hemoglobin A1C
HBsAg: HBV surface antigen
HCC: hepatocellular carcinoma
HCV: hepatitic C virus
HDL: high‐density lipoprotein (cholesterol)
HDV: hepatitis D virus
HIV: human immunodeficiency virus
MASLD: metabolic dysfunction–associated liver disease
MASLD: metabolic‐associated steatotic liver disease
NAFLD: nonalcoholic fatty liver disease
NPV: Negative Predictive Value
PPV: Positive Predictive Value
SWE: shear wave elastography
T2DM: type 2 diabetes mellitus

## 1 Introduction

Chronic hepatitis B (CHB) remains a persistent and formidable global health challenge, affecting over 240 million individuals worldwide [1]. It stands as a substantial driver of morbidity and mortality, with an increased risk of cirrhosis, hepatocellular carcinoma (HCC), and liver failure [2, 3]. Concurrently, there is a rising prevalence of encounters with patients exhibiting what was formerly termed nonalcoholic fatty liver disease (NAFLD), now recognised as metabolic dysfunction–associated liver disease (MASLD). This shift in terminology acknowledges a broader spectrum of metabolic dysregulations, including obesity, insulin resistance, and dyslipidemia, advocating for diagnostic criteria based on affirmative principles rather than exclusionary ones, transcending the nonalcoholic label [4].

The concomitant diagnosis of CHB‐MASLD has become increasingly prevalent in daily clinical practice in recent years. This intersection introduces a complex interplay, accentuating the risk of accelerated liver fibrosis progression and associated complications [5]. Consequently, managing and following up with these patients pose significant challenges.

Liver fibrosis constitutes a pivotal process in the progression to liver cirrhosis and HCC, holding substantial prognostic value in defining clinical outcomes and therapeutic regimens [6]. Traditional assessments for liver fibrosis, reliant on invasive and resource‐intensive liver biopsy, have spurred a quest for noninvasive alternatives, including serum‐based markers and advanced imaging modalities such as transient/shear wave fibroscan [7, 8]. While transient/shear wave elastography stands out for its superior diagnostic accuracy in liver fibrosis overall [9, 10], its universal applicability is hindered by factors such as limited availability and cost [11].

Extensive research has identified simple, repeatable, and reliable laboratory‐based markers [12]. Among these markers, the fibrosis‐4 index (FIB‐4) has emerged as a promising candidate for evaluating fibrosis in CHB patients, NAFLD patients, and other chronic liver diseases [13, 14]. Though the well‐established FIB‐4 score has been validated in diverse cohorts of CHB and NAFLD patients, it awaits meticulous scrutiny in the subset of patients concurrently grappling with CHB and MASLD [15].

Despite the evidence of FIB‐4 uses and validation, a knowledge gap persists regarding its performance within the intricate landscape of CHB and the burgeoning MASLD. As the prevailing prevalence of MASLD continues its upward trajectory, elucidating the diagnostic performance of these markers becomes imperative. Consequently, the current study aims to comprehensively scrutinise the diagnostic precision of FIB‐4 in predicting liver fibrosis in patients contending with CHB and CHB‐MASLD.

## 2 Methods

A prospective study was conducted, involving consecutive patients referred for clinical follow‐up due to CHB infection, defined by the presence of the HBV surface antigen (HBsAg) or evidence of viral replicative activity for a minimum of 6 months. The study spanned the years 2021–2022 and excluded individuals with HCV, HDV, or HIV coinfections. Study aims and protocols, enrolled patients provided informed consent. Subsequent interviews were conducted, including the clinic's follow‐up history. Extracted data included age, sex, ethnicity, medical history, medications, habits, and body mass index. Pertinent clinical and laboratory data on CHB infection, treatment history, coinfections, and outcomes were also meticulously compiled.

All enrolled patients underwent liver stiffness measurement using shear‐wave elastography (SWE; Supersonic Imagine, SA), performed by an experienced hepatologist with > 3 years of expertise and > 300 prior examinations. Measurements were used to stage fibrosis and dichotomised as < 8.0 kPa (minimal/mild fibrosis) or ≥ 8.0 kPa (significant fibrosis, as defined for this study). Given the heterogeneity of reported HBV cut‐offs [16], the ≥ 8.0 kPa threshold was selected based on pre‐study institutional experience, in which SWE measurements were compared with histological staging from liver biopsies in HBV patients at our centre and judged to provide a clinically balanced trade‐off between sensitivity and specificity for detecting ≥ F2 fibrosis. Hepatic steatosis was assessed on the same day by standardised abdominal ultrasonography performed by expert radiologists following a uniform institutional protocol. Steatosis was identified qualitatively and graded using the hepatorenal index (HRI), with a validated threshold of > 1.3. FIB‐4 scores were calculated from same‐day laboratory tests, including complete blood count, differential, and liver function parameters.

MASLD was defined by evidence of steatotic liver on imaging coupled with at least one cardiometabolic risk factor such as overweight (BMI > 25 kg/m^2^), dyslipidemia (HDL < 40 mg/dL, triglycerides > 150 mg/dL, or use of lipid‐lowering treatment), hypertension (blood pressure > 130/85 or use of antihypertensive medications), or dysglycemia (fasting glucose > 100 mg/dL, HA1C > 5.7%, diagnoses of type 2 diabetes mellitus (T2DM), or treatment for T2DM). Excessive alcohol consumption (defined as > 3 drinks per day) and other causes of steatotic liver disease, such as chronic steroid use or Wilson's disease, were excluded. Subsequently, distinct CHB‐alone and CHB‐MASLD groups were formed and subjected to comparative analysis.

The diagnostic performance of FIB‐4 in predicting fibrosis stage was investigated in both groups, further stratifying obese and non‐obese CHB patients. The study received approval from Hillel Yaffe's local Helsinki ethics board (ID HYMC‐0082‐19), and written consent was obtained from all participants.

### 2.1 Statistical Analysis

Continuous parameters were presented as means ± standard deviations, median, and 25%–75% percentiles, while categorical parameters were expressed as frequencies and percentages. The normal distribution of quantitative parameters was assessed using the Kolmogorov–Smirnov test. Statistical differences between minimal and significant fibrosis groups were evaluated using the *t*‐test, Mann–Whitney *U* test, and Fisher exact test for categorical parameters. ROC curves with the Youden index were employed to determine the optimal FIB‐4 score cutoff for predicting significant fibrosis in CHB‐alone and CHB‐MASLD patients. Diagnostic parameters were calculated, including Positive Predictive Value (PPV), Negative Predictive Value (NPV), sensitivity, specificity, and diagnostic performance. Pearson correlation was utilised to assess the relationship between FIB‐4 and SWE results for each group. In addition to the primary ROC analyses, a multivariable logistic regression was performed to evaluate the independent association of FIB‐4 with significant fibrosis (≥F2 by SWE), adjusting for its component variables (AST, ALT, platelet count) and relevant metabolic comorbidities (MASLD status, diabetes mellitus). Statistical analyses were performed using SPSS version 27, and significance was set at *p* < 0.05.

## 3 Results

### 3.1 Demographic Characteristics

This cohort study included 109 patients with CHB and 64 patients with CHB‐MASLD. No significant age difference was observed between the two groups (mean age 50.5 ± 12.1 in CHB vs. 49.9 ± 13.8 in CHB‐MASLD, *p* = 0.13). The distribution of gender and ethnicity was similar, with a male predominance (66% in CHB vs. 64% in CHB‐MASLD, *p* = 0.62) and a predominant Jewish ethnicity (80.7% in CHB vs. 76.5% in CHB‐MASLD, *p* = 0.17). Table 1 summarises the baseline characteristics for both groups.

**TABLE 1:** Baseline characteristics of minimal and significant fibrosis CHB groups.

Parameter *n* (%)	CHB, *n* = 109	CHB‐MASLD, *n* = 64	*p* ^a^
Age (Mean + SD)	50.5 ± 12.1	49.9 ± 13.8	*p* = 0.13
Sex (M) %	72 (66%)	41 (64%)	*p* = 0.62
Ethnicity (Jew)	88 (80.7%)	49 (76.5%)	*p* = 0.17
BMI (mean ± SD)	25.2 ± 4.0	28.5 ± 4.2	*p* = 0.028
BMI > 30 (%)	12 (11%)	19 (29.6%)	*p* < 0.01
BMI > 25 (%)	49 (45%)	57 (89%)	*p* < 0.01
HBeAg negative (%)	95 (87.1%)	55 (85.9%)	*p* = 0.52
HBeAg negative infection (%)^a^	75 (68.8%)	46 (71.8%)	*p* = 0.44
Nuclesoide/tide treatment (%)	43 (39.4%)	28 (43.7%)	*p* = 0.12
Dysglycemia (%)	11 (10%)	13 (20.3%)	*p* = 0.001
Hypertension	15 (13.7%)	11 (15.6%)	*p* = 0.21
Dyslipidemia	33 (30.3%)	26 (40.2%)	*p* = 0.02
Statin treatments	25 (22.9%)	16 (25%)	*p* = 0.45
Fibrosis markers			
FIB‐4 (mean ± SD)	1.33 ± 0.72	1.47 ± 0.53	*p* < 0.01
NLR (mean ± SD)	1.92 ± 0.98	1.86 ± 0.81	*p* = 0.12
APRI score	0.48 ± 0.17	0.75 ± 0.21	*p* < 0.01

^a^ Detailed definition provided in the methods section.

### 3.2 BMI and Metabolic Characteristics

Patients in the CHB‐MASLD group exhibited a higher mean BMI compared to the CHB group (28.5 ± 4.2 vs. 25.2 ± 4.0, *p* = 0.028). The prevalence of obesity (BMI > 30) was significantly higher in the CHB‐MASLD group (29.6% vs. 11%, *p* < 0.01). Additionally, the CHB‐MASLD group showed a higher prevalence of dysglycemia (20.3% vs. 10%, *p* = 0.001) and dyslipidemia (40.2% vs. 30.3%, *p* = 0.02).

### 3.3 Virological and Treatment Characteristics

Both groups had a high prevalence of HBeAg negativity, with no significant difference observed (87.1% in CHB vs. 85.9% in CHB‐MASLD, *p* = 0.52). Nucleoside/tide treatment rates were comparable between the CHB and CHB‐MASLD groups (39.4% vs. 43.7%, *p* = 0.12).

### 3.4 Baseline Laboratory Finding

Basic laboratory results for CHB patients in both groups are provided in Table 2.

**TABLE 2:** Basic laboratory results for CHB patients in both groups.

CHB group	CHB	CHB‐MASLD	p*
ALT	28.7 [18–52]	32.1[23.5–53]	*p* < 0.001
AST	28.1 [19–54]	30.2 [23.3–61]	*p* < 0.001
GGT	30.9 [18–65]	46.2 [29.5–85]	*p* < 0.001
Alkaline phosphatase	76.6 [58–165]	85 [69–181]	*p* < 0.001
Bilirubin total	0.79 [0.49–1.2]	0.83 [0.57–1.17]	*p* = 0.43
Albumin	4.27 ± 0.95	4.02 ± 0.47	*p* = 0.12
INR	1.05 ± 0.15	1.16 ± 0.19	*p* = 0.1
HGB	14.1 ± 1.5	14.2 ± 1.8	*p* = 0.47
MCV	87.8 ± 6.9	87.9 ± 7.3	*p* = 0.92
PLT	213 ± 61.7	191.9 ± 70.2	*p* < 0.001
NEUT	4 ± 1.60	4.20 ± 2.17	*p* = 0.30
LYMPH	2.1 ± 0.82	2.04 ± 0.78	*p* = 0.54
Creatinine	0.87 ± 0.15	0.9 ± 0.14	*p* = 0.33

*Note:* *Detailed definition provided in the methods section.

FIB‐4 index and APRI scores were significantly elevated in the CHB‐MASLD group compared to the CHB group, indicating a potential association with advanced liver fibrosis in the CHB‐MASLD subgroup.

### 3.5 Discriminative Performance of Fibrosis Markers: ROC Analysis

**FIGURE 1:**
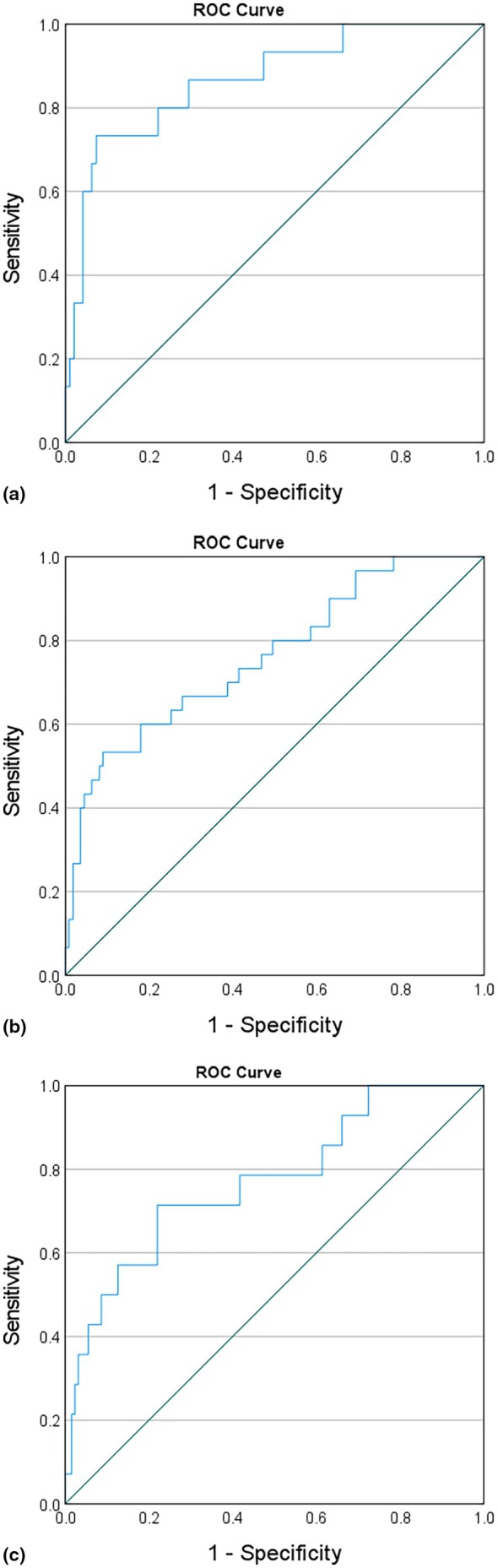
FIB‐4 ROC curve for hepatic fibrosis prediction in the overall cohort (a), obese (b), and lean (c) chronic hepatitis B patients.

A significant and linear correlation was found between the SWE score and FIB‐4 values (*r* = 0.712; *p* < 0.001). Figure 1 demonstrates the ROC curves of the FIB‐4 for detecting significant fibrosis in CHB patients. Based on this curve, the AUROC curve of FIB‐4 was 0.866 (95% confidence interval [CI] 0.63–0.89). The optimal cut‐off value of FIB‐4 for this purpose was 1.95, with a sensitivity of 81%, specificity of 77%, PPV of 92.3%, and NPV of 79.5%.

In our study cohort, sub‐classification based on BMI into categories above and below 30 did not significantly impact the predictive ability of the FIB‐4 index for detecting significant fibrosis. Despite the stratification by BMI, the FIB‐4 index maintained consistent predictive performance across both BMI groups. The overall ROC analysis and the corresponding cut‐off values indicated comparable predictive performance in individuals with a BMI ≥ 30 (AUROC 0.88, *p* < 0.01) and those with a BMI < 30 (AUROC 0.82, *p* < 0.01).

In contrast, the analysis of the FIB‐4 indexes in the cohort of individuals with CHB‐MASLD revealed a Receiver Operating Characteristic (ROC) curve with an area under the curve (AUC) of 0.61. The associated *p*‐value for the ROC curve was 0.12, indicating a non‐significant result.

### 3.6 Multivariable Analysis

In the adjusted logistic regression model, FIB‐4 remained independently associated with higher odds of significant fibrosis (adjusted OR 1.69; 95% CI 1.06–2.71; *p* = 0.028). MASLD status (OR 1.72; 95% CI 1.11–2.67; *p* = 0.015) and diabetes mellitus (OR 2.47; 95% CI 1.07–5.68; *p* = 0.034) were also independently associated with significant fibrosis, whereas AST, ALT, and platelet count were not. Collinearity diagnostics indicated acceptable levels for all covariates (all VIFs < 3).

## 4 Discussion

The simultaneous co‐existence of CHB‐MASLD is rising, yet the diagnostic performance of non‐invasive markers in this setting remains largely unexplored. This study sheds light on the potential utility of FIB‐4 in this context.

Firstly, in pursuing a practical, cost‐effective tool for fibrosis assessment in CHB patients, our study found FIB‐4 to show a commendable correlation with fibrosis prediction in this cohort. Our findings align with several previous studies suggesting a good performance of FIB‐4 in fibrosis prediction in CHB patients, albeit with varying cut‐off values [17]. Notably, a higher cut‐off value of 1.95 in our cohort was associated with the highest diagnostic performance and specificity for significant fibrosis, representing a CHB‐specific threshold that merits clinical attention. This finding is consistent with HBV‐focused evidence demonstrating improved diagnostic accuracy with higher FIB‐4 thresholds. A comprehensive meta‐analysis of 26 HBV studies (*n* = 8274) reported that cut‐offs above 2.0 yielded substantially greater specificity (0.95) and AUC (0.90) compared with lower thresholds, supporting the 2025 EASL guidance that optimal values should be context‐specific and population‐calibrated [18, 19]. In practical terms, adopting higher thresholds in CHB may reduce false positives and unnecessary downstream investigations, particularly when used in conjunction with SWE, thereby improving resource allocation without compromising clinical safety. However, while these data strengthen the case for recalibrating cut‐offs in CHB, external validation in independent HBV cohorts—ideally employing harmonised liver stiffness measurement criteria and biopsy‐correlated endpoints—remains essential before broad implementation.

Furthermore, our investigation into the performance of the FIB‐4 index revealed its robustness across the entire BMI spectrum. Comparable ROC curves for individuals with BMI above and below 30 suggest a consistent predictive ability of FIB‐4, with no significant difference in diagnostic performance between the two groups. The lack of substantial variation in sensitivity, specificity, and cutoff values across BMI strata reinforces the reliability of FIB‐4 as a predictor of significant fibrosis, irrespective of BMI status. Consistent with other recently published studies, our findings underscore the versatility and stability of the FIB‐4 index in assessing liver fibrosis across diverse BMI profiles, emphasising its potential as a reliable clinical tool for risk stratification in patients with CHB [20, 21].

However, our study uncovered a stark contrast in the performance of FIB‐4 when applied to CHB patients with MASLD. Notably, we could not identify existing studies that specifically address the performance of non‐invasive markers in this setting. The inefficacy of FIB‐4 in predicting fibrosis in this subset suggests a limitation in its applicability beyond the realm of CHB alone. This disparity in predictive performance likely arises from the complex interplay of viral and metabolic factors in the CHB‐MASLD group. While variations in disease course and outcomes were observed between CHB alone and CHB‐MASLD groups, it is crucial to note that our study was not designed to evaluate the long‐term consequences or specific characteristics of CHB‐MASLD, acknowledging the need for extended follow‐up periods to illuminate these aspects [22] comprehensively.

The absence of a well‐defined gold standard tool for fibrosis prediction in CHB‐MASLD patients poses a significant challenge and warrants further investigation. The low rate of biopsy performance led us to evaluate FIB‐4 versus SWE. However, it is imperative to assess the performance of SWE in this context. The observed concordance in CHB‐alone but not in CHB‐MASLD raises essential questions about the utility of FIB‐4 in this specific population. A comprehensive review of the existing literature underscores the necessity for future studies to identify a reliable fibrosis prediction tool tailored to the unique characteristics of CHB‐MASLD, ensuring optimal patient management and accurate risk stratification.

These findings carry significant implications for real‐life clinical practice, where the effective management of patients with CHB‐MASLD becomes increasingly challenging. The lack of validated, reliable, non‐invasive tools for fibrosis prediction emphasises the need for nuanced and individualised approaches in the clinical decision‐making process. Continued research in this area is essential to guide clinicians in optimising the care of this complex patient population, addressing the challenges posed by the interplay of viral and metabolic factors [23, 24].

The current study's strengths include its prospective design, comprehensive inclusion of virological and metabolic data, and thorough documentation and assessment of conditions to define MASLD according to the new nomenclature.

This study has several limitations. First, the absence of histological confirmation precludes definitive staging of fibrosis; liver stiffness assessment by SWE was used as the non‐invasive reference standard in line with current EASL recommendations, but this inevitably limits comparability with biopsy‐based studies. Second, the ≥ 8 kPa cut‐off applied in this study was derived from institutional biopsy–SWE calibration data and optimised for detecting ≥ F2 fibrosis in HBV patients at our center; under the 2025 EASL definitions, this threshold lies at the upper bound of “significant fibrosis” and overlaps with early “advanced fibrosis,” which may affect comparability with studies using lower thresholds. Third, hepatic steatosis was assessed by same‐day, standardised ultrasonography—the recommended first‐line modality—but its reduced sensitivity for mild steatosis may have led to underestimation of prevalence. Fourth, the MASLD subgroup size reflected its prevalence in our prospective cohort and supports real‐world generalisability but limited statistical power and increased risk of type II error. Finally, because FIB‐4 incorporates AST, ALT, and platelet count, differences in these parameters between subgroups may influence performance; however, most values were within normal to mildly elevated ranges, and multivariable analysis adjusting for these variables and metabolic comorbidities confirmed that FIB‐4 remained independently associated with significant fibrosis. These factors should be considered when interpreting the findings, and external validation in independent HBV cohorts is warranted.

## 5 Conclusion

The study contributes valuable insights into the utility of FIB‐4 for fibrosis prediction in CHB patients. It emphasises its versatility across BMI profiles but cautions against its limitations in CHB‐MASLD. The identified gaps underscore the need for further research to establish a reliable fibrosis prediction tool tailored to the unique challenges posed by the interplay of viral and metabolic factors in CHB‐MASLD patients, thereby guiding effective clinical management strategies. Future prospective multi‐centre studies in diverse populations should aim to bridge this gap by comparing FIB‐4 with liver biopsy and elucidating the course and outcomes of CHB‐MASLD.

## Author Contributions

R.T.N. and F.A.B. conceived the idea; M.H. designed the work; R.H. and T.S. analysed and interpreted the data; R.T.N. and F.A.B. drafted the work, and R.H., T.S., and M.H. revised it.

## Ethics Statement

The study received approval from Hillel Yaffe's local Helsinki ethics board (ID HYMC‐0082‐19), and written consent was obtained from all participants.

## Consent

The authors have nothing to report.

## Conflicts of Interest

The authors declare no conflicts of interest.

## Data Availability

The results/data/figures in this manuscript have not been published elsewhere, nor are they under consideration by another publisher. The datasets used and/or analysed during the current study are available from the corresponding author on reasonable request.
